# Strategies of AAV capsid engineering for targeted delivery to brain, muscle, and retina

**DOI:** 10.3389/fmolb.2025.1750807

**Published:** 2026-01-06

**Authors:** Xinyuan Xu

**Affiliations:** Postdoctoral Research Workstation, Beijing State-owned Capital Operation and Management Company Limited, Beijing, China

**Keywords:** adeno-associated virus, capsid engineering, directed evolution, machine learning, rational design

## Abstract

Adeno-associated virus (AAV) vectors are widely used for *in vivo* gene delivery to the central nervous system (CNS), muscle, and retina, but many clinically used capsids show limited potency in human tissues, necessitating high systemic doses that increase cost and toxicity risk. Here, we summarize recent capsid-engineering strategies designed to improve on-target delivery and reduce vector dose requirements. For CNS applications, receptor-informed engineering—such as capsids targeting transferrin receptor 1 (TfR1) or alkaline phosphatase (ALPL)—has produced large gains in blood–brain barrier (BBB) penetration and cross-species translation. In the retina, intravitreal (IVT) performance improves through fine-tuning of heparan sulfate proteoglycan (HSPG) interactions to facilitate inner limiting membrane (ILM) traversal, while suprachoroidal and laterally spreading subretinal vectors expand posterior-segment coverage. For muscle, next-generation myotropic and liver-detargeted capsids enable uniform skeletal and cardiac transduction at substantially lower intravenous doses. We compare directed evolution, rational design, and machine-learning (ML) approaches, highlighting how these methods increasingly converge by integrating structural hypotheses, *in vivo* selections, and multi-trait computational optimization. Quantitative benchmarks across tissues demonstrate that engineered capsids routinely deliver multi-fold improvements in potency and biodistribution relative to natural serotypes. Collectively, these advances outline a translational path toward safer, lower-dose AAV gene therapies with improved precision and clinical feasibility.

## 1 Introduction

Adeno-associated virus (AAV) is a small, non-enveloped parvovirus that is naturally replication-defective and requires helper functions for productive infection (Srivas and tava, 2016). In recombinant AAV (rAAV) vectors, the rep/cap genes are supplied in trans while the therapeutic cassette—promoter, transgene, regulatory elements—is flanked by inverted terminal repeats (ITRs); the capsid serotype/sequence largely determines tropism, intracellular trafficking, and immunogenicity (Kotterman and Schaffer, 2014; Wang et al., 2019). These properties, together with relatively low pathogenicity and long-lasting episomal expression in non-dividing cells, have made rAAV a leading *in vivo* delivery platform across liver, muscle, retina, and central nervous system (CNS) (High and Roncarolo, 2019).

Clinical-grade rAAV is produced predominantly by (i) triple transfection in HEK293 cells using plasmids encoding the vector genome, capsid, and helper functions; (ii) baculovirus–Sf9 suspension systems; or (iii) producer cell lines with stably integrated components (Clément et al., 2009). Downstream purification commonly uses ion-exchange chromatography or AAV-specific affinity resins followed by polishing and formulation (Clément et al., 2009; Liu et al., 2024).

rAAV supports three principal therapeutic strategies: gene replacement, gene editing, and RNA interference (RNAi) (Grieger et al., 2006). Gene replacement remains the dominant modality, exemplified by retinal and neuromuscular approvals where a functional cDNA restores or augments deficient protein (Dunbar et al., 2018; Maguire et al., 2008; Mendell et al., 2017; Shen et al., 2022). Gene editing uses rAAV to deliver programmable nucleases or donor templates to permanently correct mutations *in situ*; although packaging constraints and off-target risk require careful vector design, multiple liver and ocular programs are advancing (Ou et al., 2019; Maeder et al., 2019). RNAi/antisense applications include rAAV-delivered short hairpins or microRNA scaffolds to silence pathogenic transcripts in the CNS and liver; preclinical and early clinical work in mutant huntingtin suppression for Huntington’s disease has been particularly informative (Evers et al., 2018). All these efforts led to multiple rAAV gene therapy approvals, benefiting thousands of patients across diverse indications. Table 1 summarizes approved rAAV gene therapies including modality, indication, and market status, and Table 2 highlights all gene therapy programs (rAAV and non-rAAV) likely to approach biological license application (BLA) or market authorisation application (MAA) submissions in 1–2 years. These tables situate rAAV within a broader therapeutic ecosystem while underscoring its clinical footprint in systemic (e.g., muscle, liver) and local (e.g., subretinal, intracerebral) delivery.

**TABLE 1:** List of approved gene therapies.

Product	Company	Indication	Modality	Viral vector platform	Serotype/pseudotype	Price (USD)	Sales (per year)	Approval year	Region
BBM-H901	Belief BioMed	Hemophilia B	*In vivo* (intravenous)	rAAV	rAAV843	N/A	N/A	2025	China
Zevaskyn	Abeona Tx	Epidermolysis bullosa	*Ex vivo* (keratinocytes)	Gamma retroviral vector	Undisclosed	$3.1M	N/A	2025	US
Casgevy	Vertex Tx	Sickle cell disease, beta-thalassemia	*Ex vivo* (HSC*)	Nonviral	n/a	$2.2M	$10M	2023	US, UK, Bahrain, Saudi Arabia, EU, Canada, Switzerland
Elevidys	Sarepta	Duchenne muscular dystrophy	*In vivo* (intravenous)	rAAV	rAAVrh74	$3.2M	$821M	2023	US, United Arab Emirates, Qatar, Kuwait, Bahrain, Oman, Israel
Lyfgenia	Bluebird Bio	Sickle cell disease	*Ex vivo* (HSC)	Lentiviral vector (HIV1-based)	VSV-G	$3.1M	$3M	2023	US
Adstiladrin	Merck	BCG-unresponsive NMIBC	*In vivo* (intravesical)	Adenoviral vector	rAd5	$150K	$30M	2022	US
Hemgenix	UniQure	Hemophilia B	*In vivo* (intravenous)	rAAV	rAAV5	$3.5M	N/A	2022	US, EU, UK, Canada, Switzerland, Australia
Roctavian	BioMarin	Hemophilia A	*In vivo* (intravenous)	rAAV	rAAV5	$2.9M	$4M	2022	EU, US
Skysona	Bluebird Bio	Cerebral adrenoleukodystrophy	*Ex vivo* (HSC)	Lentiviral vector (HIV1-based)	VSV-G	$3.0M	$12M	2022	US
Upstaza	PTC Tx	AADC deficiency	*In vivo* (intracerebral)	rAAV	rAAV2	$4.0M	$13M	2022	EU, UK, Israel, US
Zynteglo	Bluebird Bio	Beta-thalassemia	*Ex vivo* (HSC)	Lentiviral vector (HIV1-based)	VSV-G	$2.8M	$17M	2022	US
Libmeldy	Orchard Tx	Metachromatic leukodystrophy	*Ex vivo* (HSC)	Lentiviral vector (HIV1-based)	VSV-G	$4.3M	$22M	2020	EU, UK, Switzerland, US
Zolgensma	Novartis	Spinal muscular atrophy	*In vivo* (intravenous)	rAAV	rAAV9	$2.1M	$1.2B	2019	US, EU, UK, Japan, Australia, Canada, Brazil, Israel, Taiwan, South Korea
Luxturna	Spark Tx/Roche	Inherited retinal disease (RPE65)	*In vivo* (subretinal)	rAAV	rAAV2	$850K	$51M	2017	US, EU, UK, Australia, Canada, South Korea, Japan
Glybera	UniQure	Lipoprotein lipase deficiency	*In vivo* (intramuscular)	rAAV	rAAV1	$1.0M	N/A	2012	EU (withdrawn in 2017)

Details (company, indication, modality, price, annual sales, approval year, and markets) of commercial gene therapies. Information was compiled from the U.S. FDA “Approved Cellular and Gene Therapy Products” database, official prescribing information/product labels, and ASCGT, landscape report (U.S FDA, 2025; ASGCT, 2025; Zolgensma Package Insert, 2019; Luxturna Package Insert, 2017; Adstiladrin Package Insert, 2022; Top 10 Best-Selling Gene Therapies, 2025). HSC, hematopoietic stem cells.

**TABLE 2:** List of gene therapies that are expected to submit BLA applications soon.

Product	Company	Indication	Modality	Viral vector platform	Serotype/pseudotype	Region
RP-L201	Rocket pharma	Severe leukocyte adhesion deficiency-I	*Ex vivo* (HSC)	Lentiviral vector (HIV1-based)	VSV-G	US
RGX-121	RegenX Bio	Mucopolysaccharidosis type II	*In vivo* (intracisternal)	rAAV	rAAV9	US
SEL-212	3S Bio	Refractory gout	*In vivo* (intravenous)	Nonviral	n/a	US
UX-111	UltraGenyx	Mucopolysaccharidosis Type IIIA	*In vivo* (intravenous)	rAAV	rAAV9	US
Vusolimogene oderparepvec	Replimune	Primary melanoma	*In vivo* (subcutaneous)	Oncolytic HSV-1	HSV-1	US
PRGN-2012	Precigen	Recurrent respiratory papillomatosis	*In vivo* (subcutaneous)	Adenoviral vector	Gorilla adenovector GC46	US
RP-L102	Rocket pharma	Fanconi anemia	*Ex vivo* (HSC)	Lentiviral vector (HIV1-based)	VSV-G	EU
VM202	Helixmith	Diabetic neuropathy	*In vivo* (intramuscular)	Nonviral	n/a	China
ATSN-201 (AAV.SPR)	Atsena therapeutics	X-linked Retinoschisis (XLRS)	*In vivo* (subretinal)	rAAV	rAAV.SPR	United States of America

Details (company, indication, modality, and target markets) of close-to-approval gene therapy products. Information was compiled from the ASCGT, landscape report and company press release (ASGCT, 2025; XLRS, 2025; EMA, 2025; FDA, 2025a; FDA, 2025b).

Despite success, human data increasingly show that many clinically used rAAV capsids incompletely target the intended cells/tissues, especially after systemic dosing (Shen et al., 2022). Sponsors often compensate with higher vector doses, which can amplify innate/adaptive immune responses (e.g., complement activation, hepatotoxicity, capillary-leak–like syndromes) (Li et al., 2022; Herzog, 2015; Hinderer et al., 2018) and also increase cost. rAAV vector titers are expressed as vector genomes (vg) or viral particles (vp). vg quantifies genome-containing capsids, whereas vp quantifies all capsids—including empty or partially filled particles. Because manufacturing often yields high empty-particle content, the vp:vg ratio can vary substantially, and “high dose” exposure reflects the total capsid load (vp/kg), not only the number of delivered genomes. Empty capsids increase immunogenicity, complement activation, and receptor engagement without delivering therapeutic benefit (Wright, 2014; Jarvi et al., 2024). Table 3 synthesizes publicly reported patient deaths linked to rAAV in trials and post-marketing through 12 September 2025. Importantly, the need for high systemic doses reflects not only capsid performance but also the choice of delivery route: intravenous infusion exposes vector to hepatic and splenic clearance mechanisms, whereas locoregional approaches (e.g., intravitreal, subretinal, intrathecal) achieve therapeutic transduction at far lower capsid burdens. Overall, dose requirements arise from a combination of biological and clinical constraints, including full/empty ratio, route of administration, liver transduction, and underlying disease susceptibility.

**TABLE 3:** Publicly reported patient deaths in rAAV gene therapy trials that could be possibly treatment-related.

Program/Sponsor	Indication	Vector/Route/(dose if public)	Setting	Deaths/Total patients dosed	Reported cause/Status	References
AT132 (Astellas/Audentes)	X-linked myotubular myopathy (XLMTM)	AAV8/IV (up to ∼3.5 × 10^14^ vg/kg)	Phase 1/2 (ASPIRO)	4/unknown	Severe cholestatic liver disease; four fatalities (3 in 2020, 1 in 2021); extensive safety re-analysis published	Shieh et al. (2023)
PF-06939926 (fordadistrogene) (Pfizer)	Duchenne muscular dystrophy (DMD)	AAV9/IV	Phase 1b & Phase 2 (DAYLIGHT)	2/unknown	2021: fatal SAE in non-ambulatory cohort → hold; 2024: cardiac arrest death in DAYLIGHT	Philippidis (2022a)
Elevidys/AAVrh74 programs (Sarepta)	DMD (Elevidys, post-marketing and clinical) and LGMD (SRP-9004, clinical)	AAVrh74/IV	Post-marketing and clinical	3/hundreds	FDA safety comms: 3 fatal acute liver failure (ALF) reports—2 non-ambulatory DMD (Elevidys), 1 adult LGMD in SRP-9004 trial; causality assessments evolving	U.S. Food and Drug Administration (2025)
Onasemnogene abeparvovec (Zolgensma; Novartis)	Spinal muscular atrophy (SMA)	AAV9/IV	Post-marketing	2/thousands	Acute liver failure; acknowledged by company and covered in HGT	Philippidis (2022b)
RP-A501 (Rocket)	Danon disease	AAV9/IV	Phase 2 (pivotal)	1/2	Capillary-leak syndrome; hold lifted 20 Aug 2025 with protocol changes	Reuters (2025)
CAP-002 (Capsida)	STXBP1 encephalopathy	Engineered AAV/IV	Phase 1/2	1/1	Pediatric death reported; program paused; FDA notified	Capsida Biotherapeutics (2025)
LYS-SAF302 (Lysogene)	MPS IIIA (Sanfilippo A)	AAVrh10/intracerebral	Phase 2/3 (AAVance)	1/unknown	Child death reported by sponsor during study	Philippidis (2020)
NGN-401 (Neurogene)	Rett syndrome	AAV9/intrathecal (IT); high-dose cohort 3E15 vg	Phase 1/2	1/unknown	Pediatric death after severe hyper-inflammatory syndrome at high dose; high-dose arm halted; lower-dose continues	FierceBiotech (2025)
scAAV9/JeT-GAN (NIH/UTSW)	Giant axonal neuropathy (GAN)	AAV9/IT	Phase 1 (dose-escalation)	2/14	Death 8 months post-dose related to post-op aspiration → anoxemia → cardiac arrest after spinal fusion; the other patient due to respiratory failure 60 months post dosing	Bharucha-Goebel et al. (2024)
AAV-miR-SOD1 (UMass/NEJM case series)	ALS (SOD1)	AAVrh10/IT	Case report series (NEJM)	1/2	Patient 1 developed meningoradiculitis post-infusion; later terminal respiratory arrest; autopsy enabled biodistribution/target engagement; attribution unclear	Mueller et al. (2020)

Details (company, indication, serotype, dose, route of administration, and reported causes) of publicly available patient deaths that could be linked to rAAV, treatment in clinical trials or commercial stages.

Capsid engineering efforts therefore focus on improving tissue specificity while reducing off-target uptake and immunogenicity. Strategies include (i) reducing liver tropism through surface-residue modifications or peptide insertion, (ii) lowering immunogenicity by altering capsid epitopes or minimizing empty particles to reduce complement activation, (iii) increasing genome-packaging efficiency (Mietzsch et al., 2021), (iv) enhancing species translation, (v) enriching affinity for target tissues such as CNS, retina, muscle, or kidney (Gonzalez et al., 2022), and (vi) machine learning-based approaches (Ghauri and Ou, 2023; Eid et al., 2024; Bryant et al., 2021). These approaches have generated a rapidly expanding set of engineered capsids and licensing transactions, summarized in Table 4. Together, these trends illustrate why next-generation capsids are central to reducing capsid burden (vp/kg), increasing safety, and widening the therapeutic window.

**TABLE 4:** List of licensing deals for engineered capsids.

Target tissue	Inventor	Strategy	Buyer	Deal amount	References
Muscle	Affinia Tx	Rational design	Vertex	$80M upfront/$1.6B total	News and Events (2020)
Muscle	Solid Bio	Rational design	Armatus Bio	Undisclosed	Solid Biosciences Inc (2024)
Muscle	Solid Bio	Rational design	Andelyn	Undisclosed	Solid Biosciences Inc (2025)
Muscle	Kate Tx	Directed evolution	Novartis	$1.1B acquisition	Novartis (2024)
Eye	Dyno Tx	AI	Novartis	Undisclosed	Dyno Therapeutics and Inc (2020)
Eye	Avista Tx	Directed evolution	Roche	$7.5M upfront/$1B total	Avista Therapeutics (2022)
Eye	4DMT	Directed evolution	Astellas	$20M upfront/$942.5M total	Astellas and 4D Molecular Therapeutics (2023)
Eye	Shape Tx	AI	Otsuka	$1.5B total	Otsuka (2023)
CNS	Dyno Tx	AI	Roche	$7M upfront/$220M total	JPM (2025)
CNS	Dyno Tx	AI	Roche	$50M upfront/$1.8B total	Angus Liu (2024)
CNS	Shape Tx	AI	VectorY	$1.3B total	VectorY (2025)
CNS	Capsida	Directed evolution	Abbvie	$90M upfront/$530M total	Capsida (2021)
CNS	Capsida	Directed evolution	Eli Lilly	$55M upfront/$685M total	Lilly (2023)
CNS	Voyager Tx	Directed evolution	Novartis	$54M upfront/$1.5B total	Voyager (2022)
CNS	Voyager Tx	Directed evolution	Neurocrine Bio	$175M upfront/$985M total	Voyager (2023)
CNS	Voyager Tx	Directed evolution	Novartis	$100M upfront/$1.2B total	Voyager (2024)
CNS	Sangamo Tx	Directed evolution	Genentech	$50M upfront/$1.9B total	Sangamo Therapeutics (2024)
CNS	Sangamo Tx	Directed evolution	Astellas	$20M upfront/$1.3B total	Astellas pays Sangamo (2024)
CNS	Sangamo Tx	Directed evolution	Eli Lilly	$18M upfront/$1.4B total	Sangamo The rapeutics (2025)

Details (target tissue, inventor, buyer, engineering strategy, and deal price) of reported licensing deals on engineered capsids.

## 2 Capsid engineering to improve delivery

### 2.1 CNS: species-dependent differences in blood-brain barrier (BBB) receptor usage and AAV tropism

Early systemic “BBB-penetrant” rAAVs—most notably PhP.B and the higher-potency PhP.eB—produced striking brain-wide expression after intravenous dosing in mice (Deverman et al., 2016; Chan et al., 2017), but follow-up work showed the effect was strain-restricted and hinged on the LY6A receptor on mouse brain endothelium (Huang et al., 2019; Hordeaux et al., 2018; Hordeaux et al., 2019). That receptor dependence explained the uneven performance across mouse backgrounds and the failure to translate to primates that lack LY6A, crystallizing a central lesson for the field: without a defined, conserved receptor, rodent “hits” often stall at the species boundary. The next wave moved discovery into non-human primates (NHPs) to improve human relevance. Screening in marmoset and macaque produced CAP-Mac that outforms AAV9 by 6–9-fold in NHPs (Chuapoco et al., 2023), which supported brain-wide gene transfer across several primate species (marmoset, rhesus, green monkey), albeit with age and species nuances that matter for translation. Parallel Caltech efforts iterated on the PhP.eB scaffold to yield CAP-B10 and CAP-B22, variants that show BBB crossing in marmosets and relative liver detargeting by 6–12-fold (Goertsen et al., 2022); yet infant rhesus studies reported limited potency, underlining how NHP data can still diverge across models and developmental stages. The MaCPNS1/2 family broadened the lens further: evolved in rodents but validated across rodent and primate species, these capsids robustly transduced peripheral nervous system (PNS) and CNS of marmoset and rhesus after IV dosing (4–25-fold increase over AAV9)—useful examples of cross-species performance, though again with context-specific differences (Chen et al., 2022). Interestingly, capsids selected from NHP studies did not translate well in mice, either (Stanton et al., 2023). Together, these programs advanced the state of the art while keeping the spotlight on cross-species translatability as the core risk.

Other factors contributing to this cross-species translatability issue include the evolutionary path used to select the capsid—e.g., libraries selected in C57BL/6J mice can inadvertently enrich for mouse strain–restricted solutions. A parallel effort in capsid engineering for kidney tropism showed that cross-species cycling in mouse, pig, macaque could yield more broadly compatible variants such as AAV.cc47 (Gonzalez et al., 2022). Also, post-entry events, including uncoating, episome nuclear import, and chromatinization of the delivered genome, can be species-dependent and determine how much transgene is ultimately expressed in human cells even if the vector enters the same number of nuclei (Gonzalez-Sandoval et al., 2023; Loeb et al., 2024). These findings emphasize that “translatability” spans receptor biology, evolutionary selection pressure, and nucleus-level epigenetic fate of the rAAV genome.

The most straightforward way to reduce that risk is to identify the endothelial receptor that mediates transport and design capsids around it. Voyager used receptor-mapping to show that alkaline phosphatase (ALPL) is the primary receptor for its cross-species BBB capsid VCAP-102 (Moyer et al., 2025), with direct binding to human ALPL sufficient to drive receptor-mediated transcytosis in barrier models—an instructive template for mechanistically anchored translation. In addition, another study identified LRP6 as a potential receptor mediating BBB penetration of engineered capsids (Shay et al., 2024). VCAP-102 was reported to outform AAV9 by 20–400-fold across multiple brain regions in all animal species tested.

A complementary path flips the problem: start with a known human BBB receptor and build binding into the capsid. The leading example is transferrin receptor 1 (TfR1/CD71). In 2024, the Broad Institute reported BI-hTFR1, an engineered rAAV that binds human TfR1, actively traverses human brain endothelial models, and delivers ∼40–50× higher CNS expression than AAV9 after IV dosing in human TFRC knock-in mice (Huang et al., 2024). Beyond the performance itself, the work clarified the right preclinical models (humanized TFRC knock-ins rather than wild-type mice) and provided a clean, human-anchored mechanism—receptor-mediated transcytosis—to guide optimization. Translation momentum is building around this concept: pick a human-relevant door and engineer the capsid to use it (Apertura Gene Therapy, 2025).

In addition, several groups are pursuing dose-sparing distribution via local routes to complement systemic capsids. Decades of intrathecal/cisterna magna experience show that cerebrospinal fluid (CSF) delivery can provide broad brain coverage at lower doses, and modern techniques improve the safety/consistency of these procedures (Taghian et al., 2020; Hunter et al., 2025). Building on that foundation, Latus Bio recently unveiled AAV-Ep+ and AAV-DB-3, capsids tuned for ependymal engagement and CSF-driven spread, with preclinical data in mice and NHPs and an explicit goal of reducing clinical dose requirements (Business Wire, 2025). Together, these local-route/capsid innovations offer a practical alternative when systemic dosing is constrained by pre-existing antibodies, safety margins, or biodistribution needs.

### 2.2 Retina: fine-tuning heparan sulfate binding

The intravitreal (IVT) route remains attractive for its clinical simplicity, but two anatomical/biophysical hurdles dominate: the vitreous gel and the ILM. Canonical AAV2-like capsids engage heparan-sulfate proteoglycans (HSPGs) through a cluster of basic residues on the threefold spikes—R585/R588 most critically, with contributions from R484, R487, and K532—which promotes adsorption to the vitreous/ILM and limits penetration to deeper retinal layers (Kern et al., 2003; Opie et al., 2003). Structure–function studies and mutational mapping across two decades converge on this HSPG footprint, and several groups have shown that reducing—but not abolishing—HSPG binding improves ILM traversal and outer-retina access after IVT dosing (Perabo et al., 2006). Fine-tuning basic charge near 585–588 (and neighboring positions) is therefore a central design lever for IVT-optimized capsids (Ro et al., 2025). These efforts pointed a direction for capsid engineering for improved retinal tropism, unlike muscle- and CNS-tropic capsids where the mechanism is less elucidated and strategy is more diversified.

A key proof of principle is AAV2.7m8, derived by *in vivo* directed evolution with a 10 amino acid insertion in loop 4—within the heparin/HSPG-binding domain—to enhance outer-retina transduction after IVT (Bennett et al., 2020; Dalkara et al., 2013). The original report and subsequent analyses indicate that altered HSPG interactions and physical bypass of ILM constraints underlie its improved performance relative to parental AAV2, establishing the template for modern IVT discovery campaigns. R100, the intravitreal capsid was engineered to penetrate the ILM and drive pan-retinal expression at single, low IVT doses (Calton et al., 2024). Multi-study updates in wet age-related macular degeneration (AMD) and diabetic macular edema (DME) report durable activity and favorable ocular tolerability profiles, consistent with the intended ILM traversal and broad coverage. Also, AAV2.GL and AAV2.NN use an AAV2 backbone incorporating a rationally chosen peptide insertion within surface-exposed loop IV, at a position known to modulate receptor binding and ILM traversal (Pavlou et al., 2021). In mice, AAV2.7m8 achieves ∼5–6-fold higher photoreceptor transduction than AAV2. Rationally engineered AAV2.GL and AAV2.NN further improve outer retinal expression, delivering ∼12–13-fold higher transcript levels than AAV2 and significantly outperforming AAV2.7m8 in head-to-head analyses (Pavlou et al., 2021). Mechanistically, the peptide insertion perturbs the canonical HSPG patch, consistent with the idea that reduced—rather than abolished—HSPG engagement is optimal for IVT performance. In addition, P2-V1 combines 3–5-fold higher macular/outer-retina expression over AAV2 with better evasion of neutralizing antibodies in human vitreous; phosphorylation site tweaks (e.g., YF/TV) further enhance post-entry handling and far-peripheral photoreceptor transduction (Kellish et al., 2023).

A complementary body of work explores suprachoroidal delivery, which accesses the choroid/RPE complex and can produce wide posterior-segment spread with a minimally invasive microinjector. Recent phase 2 datasets with RGX-314 (AAV8, anti-VEGF) via suprachoroidal support route feasibility and clinically meaningful anti-VEGF durability signals (Regenxbio, 2024). Beyond single programs, reviews of ocular drug delivery highlight suprachoroidal as a platform route for retina-wide gene delivery and anti-VEGF suppression, while newer data continue to refine the technique and safety profile. On the capsid for suprachoroidal delivery, AAVv128 was recently reported to achieve robust transduction across species (mouse, rabbit, NHP) with broader layer coverage after intraocular delivery, and it has shown particularly strong performance with suprachoroidal injection (several folds higher transgene expression than AAV8). These results underscore that both capsid design and route can be co-optimized for maximal retinal access (Luo et al., 2024). Similarly, coAAV-SCS-01 exhibited up to 26-fold outperformance of other capsids in targeting RPE-choroid and retinal cells of NHPs (Coave Therapeutics, 2025), making it promising for further clinical development.

Finally, several groups are pursuing geometry-aware capsids to expand reach from local injections. Atsena Therapeutics’ laterally spreading capsid, AAV.SPR, was designed to spread beyond subretinal bleb margins, enabling central-retina (foveal) exposure without foveal detachment—a clinically meaningful advantage in indications like X-linked retinoschisis (XLRS) (Couto et al., 2022). A phase 1/2 clinical trial (NCT05878860) has been initiated to assess the safety and efficacy of rAAV gene therapy for X-linked retinoschisis via subretinal administration of AAV.SPR.

For retina, the most successful IVT strategies modulate—not abolish—HSPG binding to traverse the ILM (e.g., AAV2.7m8, R100), while suprachoroidal delivery and laterally spreading subretinal vectors (AAV.SPR) offer complementary paths to pan-retinal coverage at dose-sparing exposures. Future programs should treat capsid chemistry and route engineering as a coupled system, validated across species and routes with metrics that emphasize coverage, durability, and ocular safety.

### 2.3 Muscle: dose-lowering myotropism with liver/dorsal root ganglion (DRG) detargeting

Systemic rAAV therapies for muscle diseases have demonstrated consistent biological activity—including transgene expression and restoration of missing proteins—but clinical benefit has been variable. In Duchenne muscular dystrophy (DMD), Elevidys received accelerated approval based on micro-dystrophin expression, yet the EMBARK Phase 3 trial did not meet its primary functional endpoint, and secondary outcomes showed only modest, non-significant trends. Thus, while biological activity is clear, meaningful clinical improvement remains uncertain, and high systemic doses continue to present safety challenges. In June–July 2025, the U.S. FDA disclosed investigations of fatal acute liver failure following Sarepta’s AAVrh74 platform (two deaths in non-ambulatory DMD treated with ELEVIDYS and one death in an adult with LGMD treated with SRP-9004), asked the company to suspend ELEVIDYS distribution, and placed related trials on hold while causality and risk-mitigation measures were reviewed (U.S. Food and Drug Administration, 2025). For limb-girdle muscular dystrophy (LGMD2E/R4), SRP-9003 has shown more consistent biomarker and early functional gains, and a recent Phase 3 readout reported meeting its primary endpoint in ambulatory patients. Even so, questions regarding durability and dose-related safety remain. These experiences underscore the need for next-generation muscle-tropic capsids that can achieve therapeutic benefit at lower systemic capsid burdens, reduce liver uptake, and improve safety—motivating ongoing efforts in rational engineering, directed evolution, and machine-learning-guided capsid optimization.

A strong preclinical foundation for dose reduction comes from muscle-tropic capsids discovered by *in vivo* selections: AAVMYO emerged from massively parallel *in vivo* screening as a highly myotropic AAV9 mutant that outperformed AAV9 by 11–61-fold in skeletal muscle, diaphragm, and heart of mice (Weinmann et al., 2020), while relatively sparing the liver—data that helped catalyze today’s “muscle-biased, liver-detargeted” design goals. In parallel, Tabebordbar et al. (2021) identified RGD-containing MyoAAV capsids with markedly superior skeletal muscle and cardiac transduction after IV dosing in mice and NHPs (10–50-fold higher mRNA levels than AAVrh74 in NHPs), establishing modern benchmarks for cross-species myotropism. Building directly on these efforts, Kate Therapeutics reported MyoAAV-LD variants (e.g., KT809 class) at ASGCT 2024 (PR Newswire, 2024), showing robust, uniform expression in skeletal and cardiac muscle at lower IV doses in NHPs together with reduced liver transduction, positioning the platform for clinically meaningful dose cuts versus legacy capsids.

Translation to patients is underway with proprietary clinical vectors. Solid Biosciences’ next-generation DMD program SGT-003 uses the AAV-SLB101 capsid; early 2025 readouts reported that the first six participants had detectable microdystrophin expression with an initial tolerability profile supportive of continued development, and the company outlined plans for regulatory dialogue on potential accelerated pathways (Solid Biosciences, 2025). While peer-reviewed NHP head-to-heads remain limited publicly, the data are directionally consistent with the dose reduction and liver-detargeting objective.

Across all these engineered capsids, the consensus is clear: achieving strong myotropism together with explicit liver and DRG detargeting represents the most direct route to widening the therapeutic index for systemic muscle gene therapy. For clinical translation, programs should prioritize NHP evidence of (i) uniform skeletal and cardiac muscle expression at lower total capsid burden, (ii) quantitative liver and DRG detargeting by biodistribution and histopathology, and (iii) immunosuppression strategies that remain feasible and safe in vulnerable neuromuscular populations. These considerations have become increasingly important in light of recent safety communications involving high-dose systemic administration of AAVrh74, which emphasized that serious adverse events likely arise from the combined effects of large capsid loads, strong hepatic uptake typical of AAV9-like vectors, and underlying disease fragility—rather than from any single capsid alone.

## 3 How we engineer capsids: directed evolution, rational design, and AI

**FIGURE 1:**
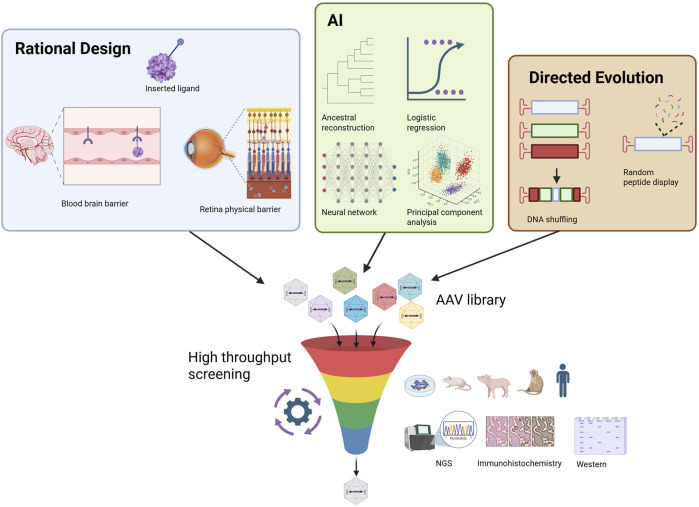
Strategies of AAV capsid engineering: directed evolution, rational design, and AI.

Directed evolution explores vast sequence space by building large, barcoded capsid libraries and selecting them *in vivo* under the relevant organ/route/species pressures, then reading out winners by deep sequencing (Figure 1). This approach routinely uncovers non-intuitive solutions that would be hard to hypothesize *a priori*. Classic examples include AAV2.7m8 for IVT administration—isolated by *in vivo* selection for outer-retina transduction after IVT, with superior performance to parental AAV2 and mechanistic follow-up confirming a loop IV insertion drives the gain of function (Dalkara et al., 2013)—and Voyager’s TRACER platform, which performed iterative selections to yield VCAP-102, a cross-species BBB-penetrant capsid later shown to use ALPL as its endothelial receptor (20–400× higher brain transfer vs. AAV9 in rodents and NHPs) (Moyer et al., 2025). Directed evolution directly optimizes against real biological barriers (e.g., the ILM, BBB, liver sinusoids) and can be executed in NHP or humanized models to mitigate species gaps. Limitations include assay cost, potential selection bias toward traits that dominate early steps (entry/trafficking), and the need to consider manufacturability and immunological constraints at design time (Ghauri and Ou, 2023).

Rational design uses structure/biophysics and mechanism to make targeted edits in receptor-interacting loops or post-entry determinants (Figure 1). Foundational work showed that substituting surface tyrosines with phenylalanine (Y to F) reduces capsid ubiquitination and proteasomal shuttling, improving intracellular trafficking and enabling dose-lowering *in vivo*; multi-YF AAV2 derivatives and extensions to other serotypes remain widely used motifs (Zhong et al., 2008; Boye et al., 2016). Moreover, AAV2i8, AAV2/AAV8 chimera, was shown to be able to efficiently transduce cardiac and skeletal muscle cells (Asokan et al., 2010), now under clinical development by AskBio. Similar logic underpins loop grafts/peptide displays to create receptor-targeted capsids (e.g., TfR1 binders for the BBB) and charge/geometry tuning at heparan-binding sites to enable ILM traversal after IVT. Rational edits can also be applied after a directed-evolution hit—to improve manufacturability, detarget off-tissue, e.g., liver, DRG, or introduce NAb-evasion features—so the two approaches are frequently combined. Rational design offers mechanistic interpretability, is relatively fast to iterate, and is ideal for multi-trait polishing (potency, detargeting, manufacturability). The flip side is search myopia—purely hypothesis-driven edits can miss emergent optima that directed evolution or machine learning (ML) can reveal.

ML uses measured sequence to function maps (from barcoded library screens) to (i) design fitter libraries enriched for viable, high-performing variants and (ii) propose *de novo* sequences that satisfy multiple traits (on-target potency, liver detargeting, manufacturability/packaging, and NAb evasion). Two representative frameworks: deep diversification (Bryant et al., 2021) trained models on a 28-aa AAV2 segment and generated >200 k variants; >110 k were viable, and many exceeded the diversity of natural serotypes—proof that ML can explore further while maintaining viability. In addition, Fit4Function (Eid et al., 2024) formalized multi-trait optimization, training on multiplexed assays to predict sequences that simultaneously meet potency and developability constraints, with experimental validation across assays/species. ML efficiently navigates combinatorial space and encodes trade-offs among traits; its main risks are assay bias and domain shift (mouse, NHP, human). Best results come when data are species-appropriate (e.g., NHP/human primary cells), include manufacturing readouts, and are iteratively refreshed with prospective validations.

A practical capsid-engineering workflow integrates rational design, directed evolution, and ML–based optimization, with each approach contributing complementary strengths. Rational design provides testable mechanistic hypotheses regarding receptor usage, surface-loop modification, physicochemical properties, and manufacturability constraints. Directed evolution enables large-scale sequence diversification and *in vivo* selection under species- and route-appropriate conditions, generating empirical evidence of tropism, potency, and detargeting. ML models trained on these sequence–function datasets can then prioritize variants that satisfy multiple traits simultaneously (e.g., potency, liver detargeting, packaging efficiency, manufacturability). Iterating these steps in human-relevant systems—such as humanized liver models, NHP BBB paradigms, and human retinal explants—has emerged as one of the most reliable routes to identifying capsids capable of achieving therapeutic expression at lower systemic doses with improved safety and CMC profiles. Recent receptor-guided examples, including ALPL or TfR1–mediated BBB penetrant vectors, demonstrate how integrating mechanistic design with *in vivo* selection and ML refinement can accelerate the development of clinically relevant candidates (Moyer et al., 2025).

Importantly, these engineering strategies are being developed alongside intensified work on immunomodulation. Severe toxicities and even deaths (Table 3) have sharpened focus on (i) transiently controlling innate and adaptive immunity at dosing (steroids, complement blockade, endothelial stabilization), (ii) targeted detargeting of high-liability organs such as liver and DRG through capsid edits, and (iii) enabling re-dosing or broadening eligibility by temporarily removing pre-existing neutralizing antibodies. IgG-cleaving proteases such as IdeS/imlifidase and IdeZ can rapidly degrade circulating anti-AAV IgG and “reset” serostatus in primates and humans, restoring transduction and potentially allowing either first-dose access for seropositive patients or true repeat dosing (Leborgne et al., 2020; Elmore et al., 2020). More recently, engineered IgM-cleaving enzymes were shown to blunt complement activation and neutralization from IgM, suggesting a path to safe re-administration and broader eligibility (Smith et al., 2024).

## 4 Discussion

### 4.1 Factors influencing systemic dose and toxicity

High-dose systemic rAAV toxicity arises from a combination of vector quality, delivery route, organ tropism, and patient susceptibility—rather than solely from limited potency of natural capsids. First, rAAV lots often contain a high proportion of empty or partially filled capsids, increasing vp/kg exposure relative to vg/kg and amplifying complement activation, receptor competition, and innate immune sensing. Second, systemic intravenous administration exposes vector to hepatic and splenic clearance pathways, whereas locoregional delivery (e.g., IVT, subretinal, intrathecal, intracisterna magna) achieves therapeutic expression at much lower total capsid loads. Third, natural serotypes such as AAV8 and AAV9 show strong hepatotropism, causing the liver to sequester a large fraction of the infused vector and increasing hepatotoxicity risk. Finally, patients with severe neuromuscular disease—including X-linked myotubular myopathy (XLMTM) and advanced DMD—often have baseline hepatic dysfunction, inflammation, and reduced physiologic reserve, making them more vulnerable to systemic stress and immune activation at doses tolerated by healthier individuals. Therefore, capsid engineering and improved manufacturing must aim to reduce capsid burden (vp/kg), detarget the liver, enhance packaging fidelity, and improve tissue specificity to achieve meaningful dose reductions and safer systemic administration.

### 4.2 Regulatory expectation and ethical considerations for engineered capsids

Regulatory expectations for engineered rAAV capsids have evolved significantly. FDA now routinely requires (i) quantitative biodistribution of novel capsids across rodents and NHPs; (ii) mechanistic justification for any receptor-targeting strategy (e.g., TfR1, ALPL) including binding affinity, cross-reactivity, and saturability; (iii) manufacturability data, because engineered capsids may alter empty:full ratios, particle stability, or genome integrity; and (iv) liver, DRG, and complement-related safety packages enriched with biomarkers (e.g., aspartate aminotransferase, alanine aminotransferase, neurofilament light chain) and dose-response curves. For systemic indications, FDA increasingly asks sponsors to propose strategies for dose minimization, including capsid potency metrics, route optimization, and predefined halting rules for liver or complement activation. These expectations place quantitative performance benchmarks—rather than qualitative tropism alone—at the center of regulatory evaluation.

Ethical considerations now prominently influence rAAV trial design. High-dose systemic rAAV administration (>1 × 10^14^ vg/kg) has been linked to acute liver failure, complement-mediated shock, thrombotic microangiopathy, and multiple patient deaths (Table 3). Ethically, this creates an obligation to (i) justify dose selection with quantitative potency data, including lower-dose efficacy in NHPs; (ii) provide a clear rationale for why alternative routes (intrathecal, intracisterna magna, regional limb infusion) or lower-dose engineered capsids cannot achieve similar outcomes; (iii) implement independent safety monitoring, real-time complement/liver biomarker surveillance, and stringent stopping rules; and (iv) design trials that minimize pediatric exposure when adult mechanistic and biodistribution data are insufficient. As engineered capsids achieve multi-fold dose reductions, their use becomes not only a scientific improvement but an ethical requirement to mitigate preventable risk.

### 4.3 Future directions

The field is converging on a pragmatic, clinically driven blueprint. First, future CNS programs are expected to favor human-anchored mechanisms (e.g., TfR1- or ALPL-mediated receptor transcytosis) over rodent-only capsids, with humanized knock-in models plus NHP biodistribution serving as the translational gatekeepers (Shay et al., 2024; Opie et al., 2003; Perabo et al., 2006; Huang et al., 2023). Second, capsid optimization is no longer single-objective; potency in the target tissue, detargeting of high-liability organs, manufacturability/packaging quality, and resistance to prevalent neutralizing antibodies are being co-optimized from the very first design cycle (Tabebordbar et al., 2021; PR Newswire, 2024; Solid Biosciences, 2025; Zhong et al., 2008; Boye et al., 2016). Third, dose itself is now the most important parameter: the most important deliverable for next-generation capsids is multi-fold dose reduction at equal or better efficacy, because that directly widens the therapeutic window, lowers manufacturing cost per patient, and reduces the likelihood of catastrophic systemic toxicities (Shay et al., 2024; Huang et al., 2024; Apertura Gene Therapy, 2025; Taghian et al., 2020; Weinmann et al., 2020; Tabebordbar et al., 2021; PR Newswire, 2024; Solid Biosciences, 2025; Zhong et al., 2008; Boye et al., 2016). Fourth, patient access and durability will increasingly hinge on immune management and re-dosing: IgG- and IgM-cleaving biologics and complementary immunomodulatory regimens are being advanced precisely to (i) open trials to patients who are currently excluded by pre-existing anti-AAV antibodies, (ii) permit follow-on dosing to boost expression as children grow, and (iii) improve safety by reducing complement-driven acute reactions (Leborgne et al., 2020; Elmore et al., 2020; Smith et al., 2024). Finally, looking 5–10 years ahead, we anticipate three transformative shifts: (i) receptor-defined BBB shuttles making systemic neurology gene therapy viable at doses an order of magnitude below today’s benchmarks; (ii) office-based intravitreal or suprachoroidal ocular gene therapy that no longer requires subretinal surgery; and (iii) muscle-predominant, liver-detargeted capsids that unlock safe systemic treatment for neuromuscular and cardiomyopathic diseases. Together, these advances point toward an rAAV gene therapy landscape that is more precise, lower dose, more redosable, and ultimately more accessible to patients.
